# Role of PKC and CaV1.2 in Detrusor Overactivity in a Model of Obesity Associated with Insulin Resistance in Mice

**DOI:** 10.1371/journal.pone.0048507

**Published:** 2012-11-07

**Authors:** Luiz O. Leiria, Carolina Sollon, Marina C. Calixto, Letícia Lintomen, Fabíola Z. Mónica, Gabriel F. Anhê, Gilberto De Nucci, Angelina Zanesco, Andrew D. Grant, Edson Antunes

**Affiliations:** 1 Department of Pharmacology, Faculty of Medical Sciences, University of Campinas (UNICAMP), Campinas, São Paulo, Brazil; 2 Department of Physical Education, Institute of Bioscience, University of São Paulo State (UNESP), Rio Claro, São Paulo, Brazil; 3 Wolfson Centre for Age-Related Diseases, King’s College, London, United Kingdom; College of Tropical Agriculture and Human Resources, University of Hawaii, United States of America

## Abstract

Obesity/metabolic syndrome are common risk factors for overactive bladder. This study aimed to investigate the functional and molecular changes of detrusor smooth muscle (DSM) in high-fat insulin resistant obese mice, focusing on the role of protein kinase C (PKC) and Ca_v_1.2 in causing bladder dysfunction. Male C57BL/6 mice were fed with high-fat diet for 10 weeks. *In vitro* functional responses and cystometry, as well as PKC and Ca_v_1.2 expression in bladder were evaluated. Obese mice exhibited higher body weight, epididymal fat mass, fasting glucose and insulin resistance. Carbachol (0.001–100 µM), α,β-methylene ATP (1–10 µM), KCl (1–300 mM), extracellular Ca^2+^ (0.01–100 mM) and phorbol-12,13-dibutyrate (PDBu; 0.001–3 µM) all produced greater DSM contractions in obese mice, which were fully reversed by the Ca_v_1.2 blocker amlodipine. Cystometry evidenced augmented frequency, non-void contractions and post-void pressure in obese mice that were also prevented by amlodipine. Metformin treatment improved the insulin sensitivity, and normalized the *in vitro* bladder hypercontractility and cystometric dysfunction in obese mice. The PKC inhibitor GF109203X (1 µM) also reduced the carbachol induced contractions. PKC protein expression was markedly higher in bladder tissues from obese mice, which was normalized by metformin treatment. The Ca_v_1.2 channel protein expression was not modified in any experimental group. Our findings show that Ca_v_1.2 blockade and improvement of insulin sensitization restores the enhanced PKC protein expression in bladder tissues and normalizes the overactive detrusor. It is likely that insulin resistance importantly contributes for the pathophysiology of this urological disorder in obese mice.

## Introduction

Metabolic syndrome is a highly prevalent public health problem that is defined by the coexistence of central obesity, insulin resistance, glucose intolerance, dyslipidemia and arterial hypertension [1]. Metabolic syndrome increases the risk of developing type II diabetes and cardiovascular diseases [2]. Insulin resistance is the central feature of metabolic syndrome, whereby target tissues fail to respond efficiently to normal concentrations of insulin. Obesity is the main etiological factor that predisposes to insulin resistance and vascular diseases [3]–[5].

Along with the dramatic increase in the incidence of diabetes, obesity and metabolic syndrome, there has been a parallel increase in urological complications [6]. Obesity and metabolic syndrome have been established as common risk factors for lower urinary tract symptoms (LUTS) including overactive bladder and urinary incontinence [7]–[9]. Overactive bladder is a highly prevalent condition that affects millions of people worldwide with a profound effect on quality of life and considerable costs to health care systems. The third National Health and Nutritional Examination Survey (NHANES III) showed that an increase in body mass index (BMI) in people over 25 years of age is positively correlated with LUTS, and men with a larger waist circumference (>102 cm) are more likely to exhibit LUTS [10]. However, few studies have explored the mechanisms of bladder dysfunction in metabolic syndrome / diabetes. Male Wistar rats fed with 60% fructose-enriched chow for 6 weeks became overweight, hyperinsulinemic and hyperglycemic, and exhibited unstable premicturition bladder contractions that were suggestive of detrusor overactivity [11]. Female Wistar rats treated with the same high-fructose diet for prolonged periods (24 weeks) showed no increase in body weight, although insulin resistance was observed [12]. Bladders from these animals showed markedly lower bladder contractions to electrical-field stimulation (EFS), carbachol and KCl *in vitro*, along with higher contractions to ATP. A cystometric study carried out in conscious female obese Zucker rats revealed decreased voiding frequency and non-voiding contractions in both obese non-diabetic and obese diabetic animals, suggesting that chronic obesity itself reduces the activity of the bladder, with no significant roles in voiding outcomes due to diabetes [13]. Although human obesity is mostly derived from consumption of high-fat diets combined with low expenditure of energy [14], only a single experimental study has focused on bladder activity in high-fat diet-induced obesity. Male Sprague-Dawley rats fed with a hyperlipidemia diet for 24 weeks gained more weight and showed an increase in voiding and non-voiding bladder contractions as evaluated by cystometry, suggesting bladder overactivity [15]. Urinary bladder dysfunction during obesity has thus been poorly studied, with conflicting findings that may be due to differences in the strain and sex of test animals, and experimental paradigm.

Protein kinase C (PKC) isoforms have been established as important regulators of lipid-induced insulin resistance [16]. Defects in the diacylglycerol (DAG)-PKC pathway in skeletal muscle and adipocytes are implicated in the insulin-resistant states of obesity and type II diabetes, as observed in animals [17], [18] and humans [19]. Under physiological conditions, coupling of muscarinic receptors (mAChR) to Ca_v_1.2 L-type Ca^2+^ channels in pig and mice bladder has recently been shown to involve an atypical signaling cascade involving associated PKC and Ca_v_1.2 channels [20]. In the present study we have used a model of metabolic syndrome, in which mice on a high-fat diet develop obesity and insulin resistance, to further investigate the functional and molecular changes in the urinary bladder in this condition. Since elevated Ca^2+^ entry via Ca_v_1.2 L-type Ca^2+^ channels plays a major role in the altered detrusor contractions observed under diabetic conditions [21], we have also focused on the role of PKC-mediated extracellular Ca^2+^ influx through Ca_v_1.2 Ca^2+^ channels to bladder dysfunction and its relationship with insulin resistance in the high-fat fed obese mice.

## Materials and Methods

### Animals

All animal procedures and experimental protocols are in accordance with the Ethical Principles in Animal Research adopted by the Brazilian College for Animal Experimentation (COBEA) and were approved by the institutional Committee for Ethics in Animal Research/State University of Campinas (CEEA-UNICAMP, protocol number 2067-1). Four-week-old male C57BL6/J mice were provided by the Central Animal House Services of State University of Campinas (UNICAMP).

### Diet-induced Obesity and Treatments

The animals were housed three per cage on a 12 h light–dark cycle, and fed for 10 weeks with either a standard chow diet (carbohydrate: 70%; protein: 20%; fat: 10%) or a high fat diet that induces obesity (carbohydrate: 29%; protein: 16%; fat: 55%), as previously described [22], [23].

Lean and obese mice were further divided into two additional groups, namely, animals treated chronically with either the L-type Ca^2+^ channel blocker amlodipine (25 mg/kg/day, given in the drinking water from the 7^th^ to the 10^th^ week) [24] or the insulin sensitizing agent metformin (300 mg/kg^/^day, given by gavage from the 8^th^ to the 10^th^ week) [25]. Tail-cuff pressure was evaluated in all groups, adapted to mice.

### In vivo Insulin Sensitivity

After 6 h fasting, systemic insulin sensitivity was analyzed by the Insulin Tolerance Test (ITT) which was performed. Tail blood samples were collected before (0 min) and at 5, 10, 15, 20, 25 and 30 min after an intraperitoneal injection of 1.00 U/Kg of regular insulin (Novolin R, NovoNordisk, Bagsvaerd, Denmark). Glucose concentrations were measured using a glucometer (ACCUCHEK Performa; Roche Diagnostics, Indianapolis, IN, USA) and the values were used to calculate the constant rate for blood glucose disappearance (Kitt), based on the linear regression of the neperian logarithm of glucose concentrations obtained from 0 to 30 min of the test. Kitt was calculated using the formula 0.693/(t1/2)×−1×100.

### In vitro Functional Assays

Mice were anesthetized with isoflurane, and urinary bladders removed and sectioned horizontally at the level of the ureters. Two longitudinal detrusor smooth muscle (DSM) strips with intact urothelium were obtained from each bladder. Strips of DSM were mounted in 10 ml organ baths containing Krebs-Henseleit solution with the following composition (mM): 117 NaCl, 4.7 KCl, 2.5 CaCl_2_, 1.2 MgSO_4_, 1.2 KH_2_PO_4_, 25 NaHCO_3_ and 11 glucose, pH 7.4, at 37°C and bubbled with a gas mixture of 95% O_2_ and 5% CO_2_. Changes in isometric force were recorded using a Power Lab v.7.0 system (Colorado Springs, CO, USA). The resting tension was adjusted to 0.5 g at the beginning of the experiments. The equilibration period was 60 min and the bathing medium was changed every 15 min.

Nonlinear regression analysis to determine the pEC_50_ was carried out using GraphPad Prism (GraphPad Software, San Diego, CA, USA) with the constraint that Φ = 0. All concentration–response data were evaluated for a fit to a logistics function in the form:
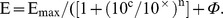



*E* is the maximum response produced by agonists; *c* is the logarithm of the EC_50_, the concentration of drug that produces a half-maximal response; *x* is the logarithm of the concentration of the drug; the exponential term, *n*, is a curve-fitting parameter that defines the slope of the concentration–response line and Φ is the response observed in the absence of added drug. The values of pEC_50_ data represent the mean ± S.E. Maximal response (E_max_) data were normalized to the wet weight of the respective urinary bladder strips, and the values of E_max_ were represented by mN per milligram wet weight.

### Contractile Responses to Carbachol, α,β-methylene ATP, KCl, Extracellular CaCl_2_ and phorbol-12,13-dibutyrate (PDBu) in Mice DSM

Cumulative concentration-response curves to the mAChR agonist carbachol (0.001–100 µM), the PKC activator PDBu (0.001–3 µM) and KCl (1–300 mM) in DSM strips were performed. Contractile response to the P2X1 purinergic agonist α,β-methylene ATP were obtained through non-cumulative addition of three different drug concentrations (1, 3 and 10 µM).

Cumulative concentration-response curves to extracellular CaCl_2_ (0.01–30 mM) in depolarizing conditions were constructed. The strips were prepared and mounted in 10 ml organ baths containing Krebs-Henseleit Ca^2+^-free solution containing EGTA (1 mM) to sequester Ca^2+^ ions, and cyclopiazonic acid (CPA, 1 µM) to deplete sarcoplasmic reticulum Ca^2+^ stores. Next, bath solution was removed and replaced by Krebs-Henseleit Ca^2+^-free solution containing KCl (80 mM) and CPA (1 µM). After an equilibration period of 15 min, the cumulative curve to CaCl_2_ was constructed [26].

The concentration-response curves to carbachol, KCl, PDBu and extracellular CaCl_2_ in DSM strips obtained from both lean and obese mice were performed in the absence and in the presence of either the L-type Ca^2+^ channel blocker amlodipine (3 µM, 30 min) or antidiabetic agent metformin (1 µM, 30 min). Concentration-response curves to carbachol were also performed in the presence of the PKC inhibitor GF109203X (1 µM, 30 min). Contractile responses to PDBu was also evaluated in a Ca^2+^-free Krebs buffer medium. Contractile responses to these agents were also evaluated in DSM strips from lean and obese mice treated chronically with amlodipine (25 mg/kg/day for 21 days) or metformin (300 mg/kg/day, 14 days).

### Electrical-field Stimulated-induced DSM Contractions

Frequency-response curves (1–32 Hz) were elicited by stimulating the tissues for 10 s with pulses of 1 ms width at 80 V, with 3 min interval between stimulations. Subsequently after 30 min incubation periods, frequency-response curves were repeated in the presence of the non-selective mAChR antagonist atropine (1 µM, 30 min) and the P2X receptor blocker PPADS (30 µM, 30 min), to confirm the mediation by mAChR and P2X receptor activation. Frequency-response curves were also performed in the presence of the voltage-gated sodium channel blocker tetrodotoxin (1 µM, 30 min) to confirm the neurogenic nature of the contractions.

### Cystometry

Mice were anaesthetized with an intraperitoneal injection of urethane (1.8 g/kg). A 1 cm abdominal incision was made, the bladder was exposed and a butterfly cannula (25 G) was inserted into the bladder dome. The cannula was connected to a three-way tap, one port of which was connected to a pressure transducer and the other to the infusion pump through a catheter (PE50). Before starting the cystometry, the bladder was emptied via the third port. Continuous cystometry was carried out by infusing saline into the bladder at a rate of 0.6 ml/h. The following parameters were assessed: Threshold pressure (TP; the intravesical pressure immediately before micturition); Post-void pressure (PVP; the intravesical pressure immediately after micturition); Peak pressure (PP; the peak pressure reached during micturition); Capacity (CP; the volume of saline needed to induce the first micturition); Compliance (CO; the ratio of CP to TP); Frequency of voiding contractions (VC) and frequency of non-voiding contractions (NVCs). NVCs were defined as spontaneous bladder contractions greater than 4 mmHg from the baseline pressure that did not result in a void. Bladders from mice used in the cystometry were not used in the other experiments.

### Western Blotting Detection of PKC and Ca_v_1.2 Calcium Channels in Urinary Bladder

Bladder tissues were homogenized in a SDS lysis buffer with a Polytron PTA 20S generator (model PT 10/35; Brinkmann Instruments, Inc., Westbury, NY) operated at maximum speed for 30 sec and centrifuged (12,000×*g*, 4°C, 20 min) to remove insoluble material. Protein concentrations of the supernatants were determined by the Bradford assay, and equal amount of protein from each sample (50 µg) was treated with Laemmli buffer containing dithiothreitol 100 mM. Samples were heated in a boiling water bath for 15 min and resolved by SDS-PAGE. Electrotransfer of proteins to nitrocellulose membrane was performed for 60 min at 15 V (constant) in a semi-dry device (Bio-Rad, Hercules, CA, USA). Nonspecific protein binding to nitrocellulose was reduced by pre-incubating the membrane overnight at 4°C in blocking buffer (0.5% non-fat dried milk, 10 mM Tris, 100 mM NaCl, and 0.02% Tween 20). Detection using specific antibodies, HRP-conjugated secondary antibodies, and luminol solution was performed, as described previously [27]. Anti-PKC (ab59363), anti-DHPRα1 subunit (ab58552) antibodies were obtained from AbCam Technology (Cambridge, England, UK)and and anti GAPDH (SC25778) was from Santa Cruz Biotechnologie (Santa Cruz, CA, USA. Densitometry was performed using the Scion Image software (Scion Corporation, Frederick, MD) and results represented as the ratio of the density of the PKC / Ca_V_1.2-α1 band to the density of the GAPDH band.

### Drugs

Urethane, metformin, carbachol, pyridoxalphosphate-6-azophenyl-2’,4’-disulfonic acid (PPADS), α,β-methylene ATP, atropine, amlodipine, tetrodotoxin, phorbol-12,13-dibutyrate (PDBu) and cyclopiazonic acid were obtained from Sigma (St. Louis, MO, USA).

### Statistical Analysis

Data are expressed as mean ± SEM of n experiments. In the cumulative concentration and frequency-response curves data were expressed as mean of the contraction in mN/mg of wet strip weight ± SEM of *n* experiments. The program Instat (GraphPad Software) was used for statistical analysis. One-way analysis of variances (ANOVA) followed by a Tukey test was used in all groups, and p<0.05 was accepted as significant.

## Results

### Body Weight, Epididymal Fat Mass and Insulin Sensitivity

**Figure 1:**
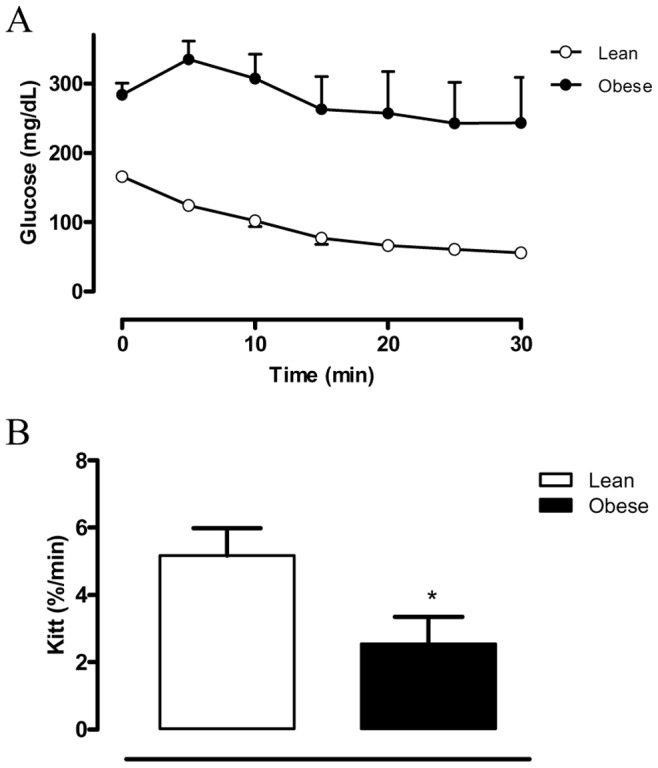
Insulin tolerance test (ITT): (A) insulin sensitivity test after intraperitoneal injection of insulin (1.00 U/kg body wt) was performed on lean and obese groups. Blood samples were collected from the tail at indicated time points and analyzed for glucose concentration; (B) the constant rate for blood glucose disappearance (Kitt), based on the linear regression of the neperian logarithm of glucose concentrations, obtained from indicated time points. Results are expressed as mean ± SEM from 5–7 animals in each group.

Obese mice exhibited a significant increase in body weight and epididymal fat mass compared with lean mice (p<0.001; Table 1). Bladder weight was not significantly modified between lean and obese groups. Fasting glucose levels were increased by 70% in obese group (p<0.001; Table 1), whereas insulin sensitivity was markedly reduced when compared with lean mice (p<0.05), as evidenced by the curve representing glucose decay and Kitt values (Figure 1A and B). Tail-cuff pressure was not modified in obese compared with lean group (84±3.4 and 86±3.3 mmHg, respectively**)**.

**Table 1:** Body weight, epididymal fat mass, bladder weight and glucose levels in lean and high-fat fed obese mice, treated or not with metformin (300 mg/kg/day, 14 days).

	Lean	Lean + Metformin	Obese	Obese + Metformin
Body weight (g)	28.5±0.3 *(9)*	28.4±0.3 *(10)*	45.7±1.1*** *(9)*	43.7±0.9*** *(10)*
Epididymal fat mass (g)	0.25±0.01 *(9)*	0.39±0.3 *(10)*	1.71±0.08*** *(9)*	1.86±0.1*** *(10)*
Bladder weight (mg)	24.9±4.1 *(6)*	25.7±0.8 *(7)*	27.9±6.9 *(6)*	27.1±0.9 *(7)*
Glucose (mg/dl)	139±6.6 *(9)*	166±10 *(5)*	238±12*** *(10)*	162±5.2# *(5)*

Data represent the means ± SEM for 5–10 mice.

*** p<0.001 compared with lean group;

# p<0.001 compared with respective obese group.

### In vitro Functional Assays

**Figure 2:**
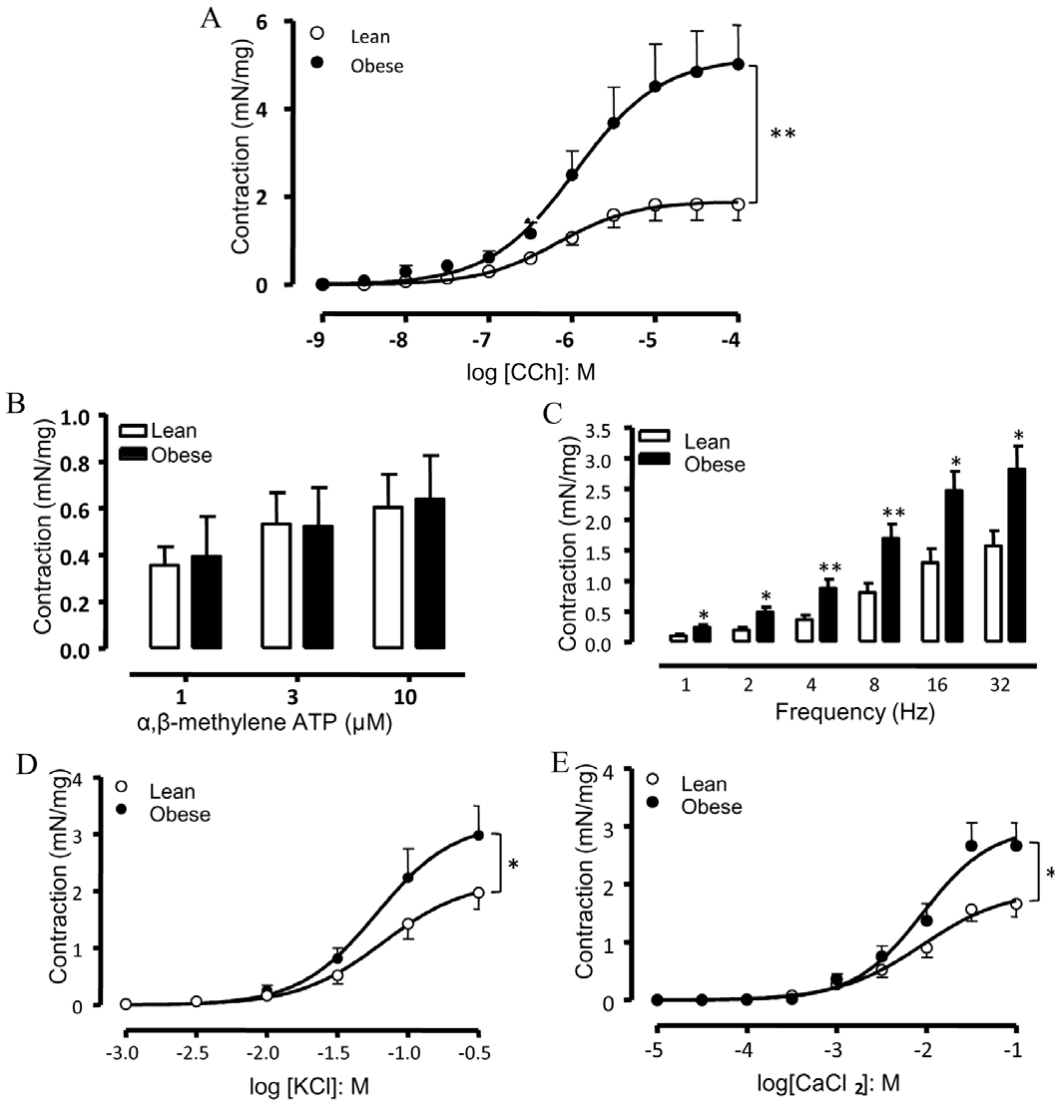
Detrusor smooth muscle contraction in response to the muscarinic agonist carbachol (A), the purinergic P2X agonist α,β-methylene-ATP (B), electrical-field stimulation (C), KCl (D) and CaCl_2_ (E) in bladder strips from control and obese mice. Data represent the mean ± SEM for 6 to 7 mice each group. * p<0.05, ** p<0.01 compared with control group.

Figure 2A shows that the mAChR agonist carbachol (1 nM – 100 µM) produced concentration-dependent contractions in strips of isolated DSM with a maximal response (E_max_) greater in the obese (p<0.01) than the lean group (5.02±0.89 and 1.83±0.36 mN/mg, respectively; n = 6–7). No significant differences for the pEC_50_ values for carbachol were found between the lean and obese groups (6.23±0.09 and 6.11±0.06, respectively).

Contractile responses to the P2X receptor agonist α,β-methylene ATP (1 – 10 µM) did not significantly differ between control and obese groups (n = 6–7; Figure 2B).

Electrical-field stimulation (EFS; 1–32 Hz) produced frequency-dependent DSM contractions in both groups, which were greater in obese mice at all frequencies employed (Figure 2C *;* n = 6). Pre-treatment of DSM preparations with the mAChR antagonist atropine (1 µM) together with the purinergic receptor blocker PPADS (30 µM) markedly reduced (p<0.001) the EFS-induced contractions in both lean (E_max_: 1.57±0.25 and 0.37±0.09 mN/mg for untreated and treated preparations, respectively; n = 6) and obese mice (E_max_: 2.82±0.38 and 0.93±0.19 mN/mg for untreated and treated preparations, respectively; n = 6). Incubation with the voltage-gated sodium channel blocker tetrodotoxin (1 µM) almost abolished the EFS-elicited contractions at all frequencies tested (n = 4, data not shown).

Cumulative concentration-response curves to the receptor-independent agents KCl and CaCl_2_ were also obtained in DSM strips from obese and lean mice (Figure 2 D and E). Potassium chloride (KCl; 1 – 300 mM; n = 6–7) and CaCl_2_ (0.01 – 100 mM; n = 5–6) produced concentration-dependent DSM contractions with E_max_ significantly greater in obese mice for both agents (p<0.05) compared with the lean group (Figure 2D and E). No differences at the pEC_50_ levels for KCl and CaCl_2_ were found between lean (1.25±0.07 and 2.19±0.08, respectively) and obese groups (1.36±0.07 and 2.14±0.06, respectively).

### Effect of Ca_v_1.2 L-type Ca^2+^ Channels Blockade by Amlodipine

**Figure 3:**
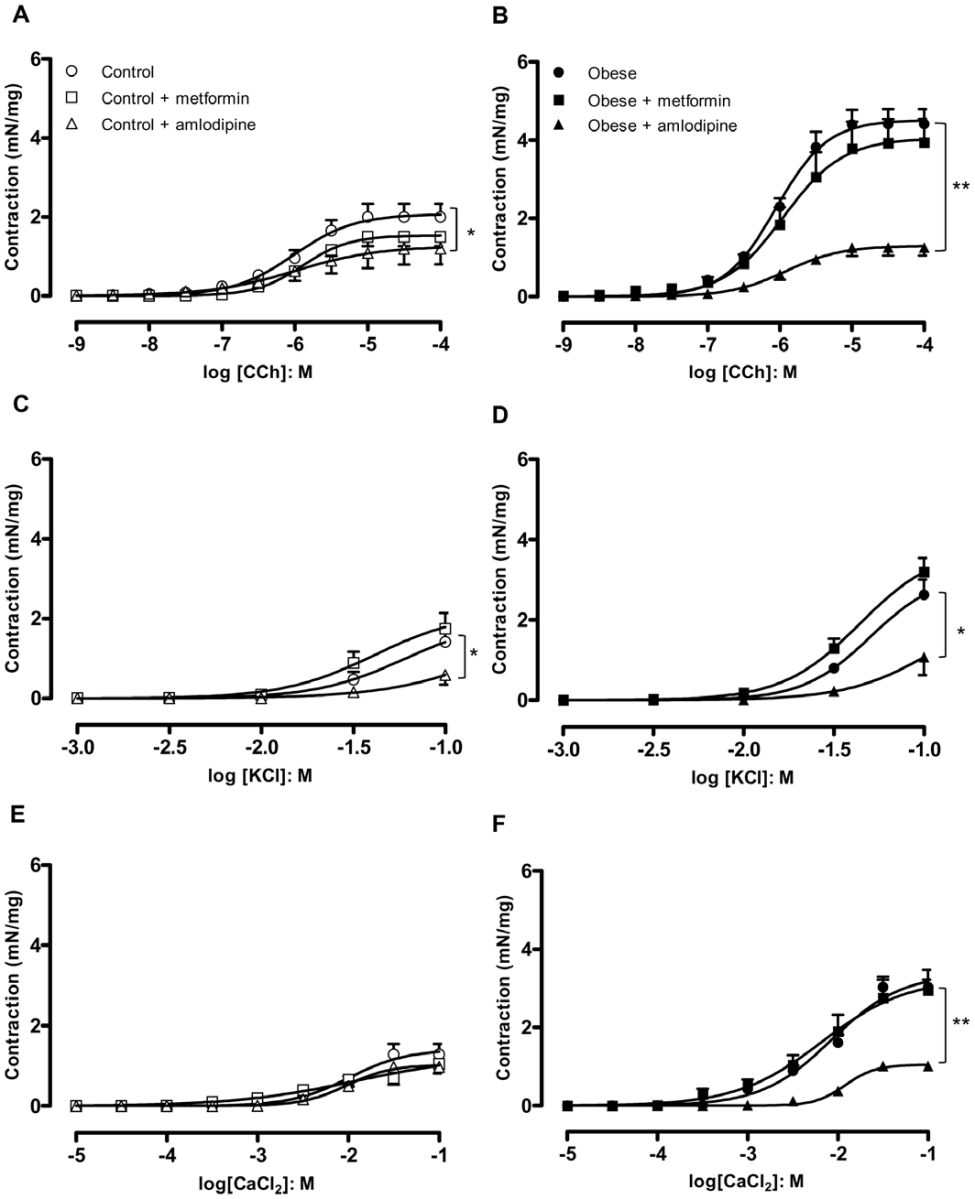
Cumulative concentration-response curves to carbachol (A and B), KCl (C and D) and CaCl_2_ (E and F) in the presence of *in vitro* pre-incubated metformin (1 µM) or amlodipine (3 µM) in detrusor smooth muscle from lean and obese mice. Data represent the mean ± SEM for 4 to 7 mice each group. * p<0.05, ** p<0.01 compared with untreated group.

Since KCl and CaCl_2_-induced contractions were enhanced in strips taken from obese DSM, we hypothesized that an increase in Ca^2+^ entry through L-type voltage-operated Ca^2+^ channels is likely to play a key role in the overactive DSM. *In vitro* incubation of DSM with the dihydropyridine calcium channel blocker amlodipine (3 µM; n = 4–5) nearly normalized the enhanced contractile responses (E_max_) to carbachol in obese mice, with a small but significant inhibition of contraction in the lean mice (Figure 3 A and B). Similarly to carbachol, the enhanced contractile responses to KCl (Figure 3 C and D; n = 5–7) and CaCl_2_ (Figure 3 E and F; n = 4–5) in obese mice were prevented by *in vitro* incubation with amlodipine.

**Figure 4:**
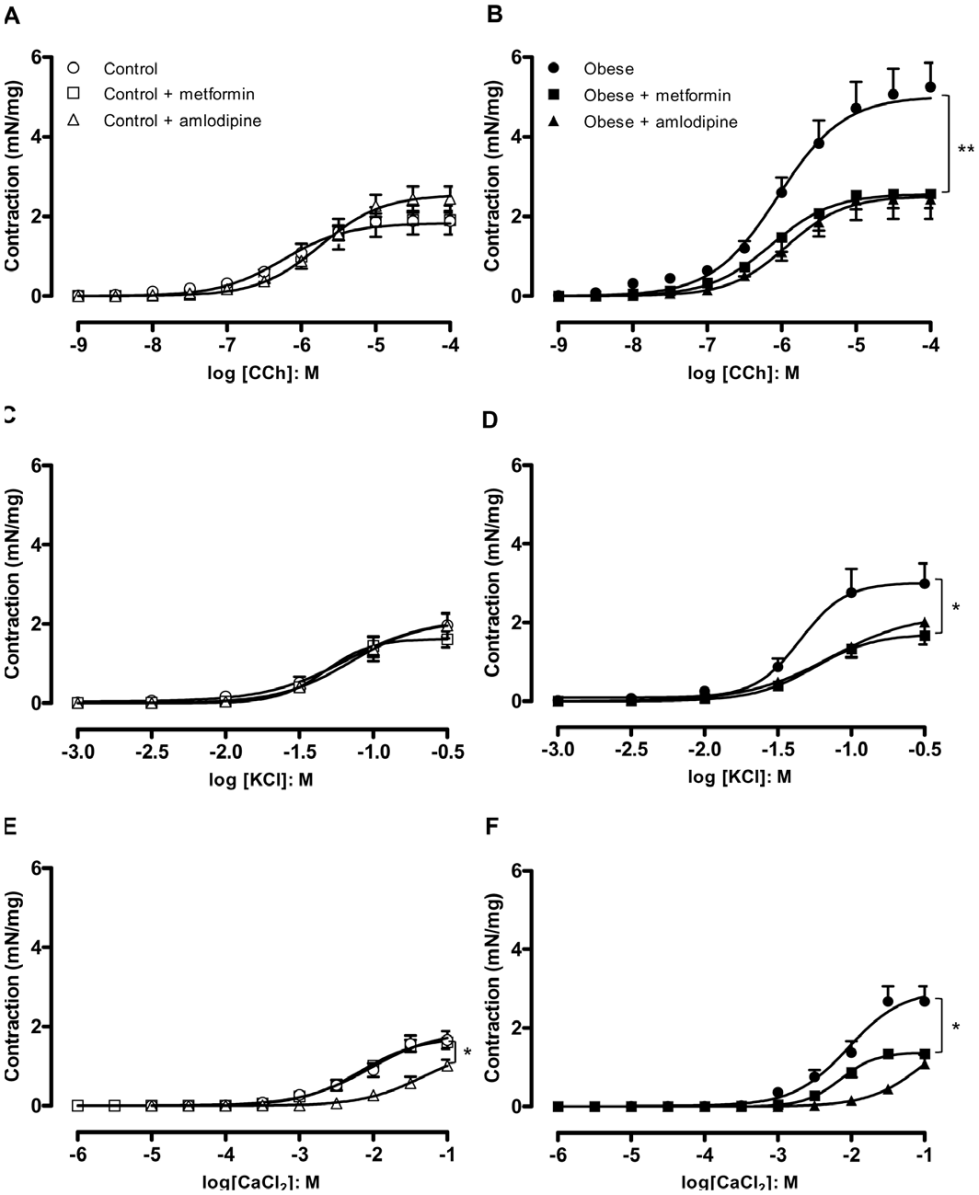
Cumulative concentration-response curves to carbachol (A and B), KCl (C and D) and CaCl_2_ (E,F) in detrusor smooth muscle from lean and obese mice chronically treated with metformin (300 mg/kg/day, 14 days) or amlodipine (25 mg/kg/day, 21 days). Data represent the mean ± SEM for 5 to 11 mice each group. * p<0.05, ** p<0.01 compared with untreated group.

In separate groups, obese and lean mice were treated orally with amlodipine (25 mg/kg/day, 21 days), and contractile responses of DSM strips to carbachol (n = 6–7), KCl (n = 5–10) and CaCl_2_ (n = 5–11) were obtained. Long-term amlodipine administration prevented the enhancement of contractions to carbachol (Figure 4 A and B), KCl (Figure 4 C and D) and CaCl_2_ (Figure 4 E and F) in obese mice. In lean group, oral amlodipine did not significantly alter the contractile responses to carbachol and KCl, but significantly reduced the CaCl_2_-induced DSM contractions.

### Role for Insulin Resistance in the Overactive DSM in Obese Mice

Chronic treatment with the anti-hyperglycemic agent metformin (300 mg/kg/day, 14 days) did not significantly affect body weight and epididymal fat mass in obese or lean mice (Table 1, n = 10). However, metformin treatment normalized the lower insulin sensitivity seen in obese mice, restoring the Kitt to control values (5.17±0.81, 4.68±1.23, 2.54±0.80 and 5.76±0.38 % / min for lean untreated, lean treated, obese untreated and obese treated, respectively; n = 4 – 6), as well as the fasting blood glucose values (166±10 and 162±5.2 mg/dL for lean and obese treated, respectively; n = 5).

Chronic metformin treatment had no significant effect on DSM contractions in lean mice (Figure 4 A, C and E). However, the increase in DSM contractions to carbachol (Figure 4B), KCl (Figure 4D) and CaCl_2_ (Figure 4F) in obese mice was suppressed by chronic metformin treatment (n = 6 – 11). In contrast, *in vitro* incubation of DSM strips with metformin (1 µM, 30 min) had no effect on DSM contractions in lean or obese mice (Figure 3).

### Cystometric Studies

**Figure 5:**
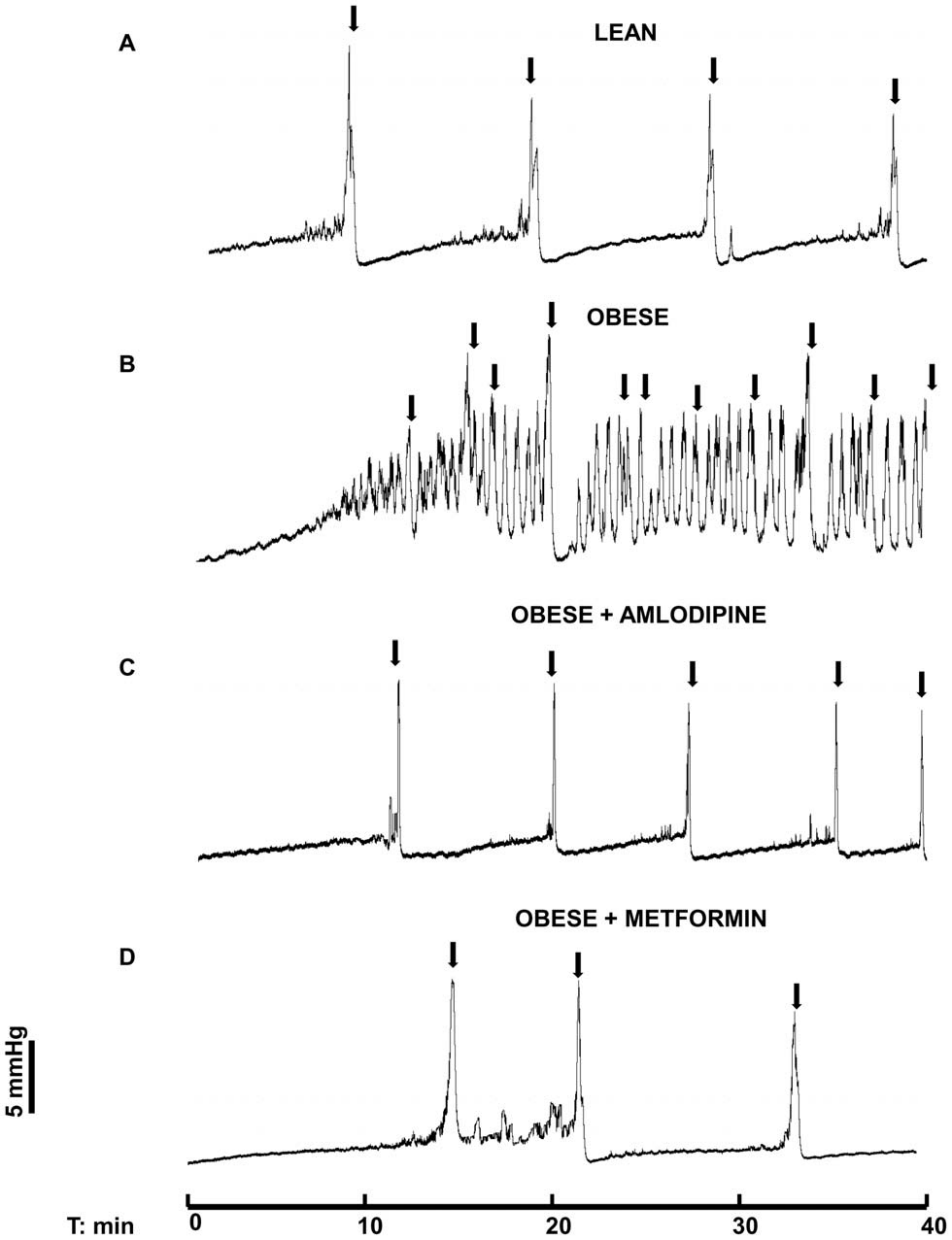
Representative cystometric recordings from lean (A), obese (B), obese treated with amlodipine (C) and obese treated with metformin (D) mice. Arrows in the cystometric trace indicate the micturition peaks.

**Figure 6:**
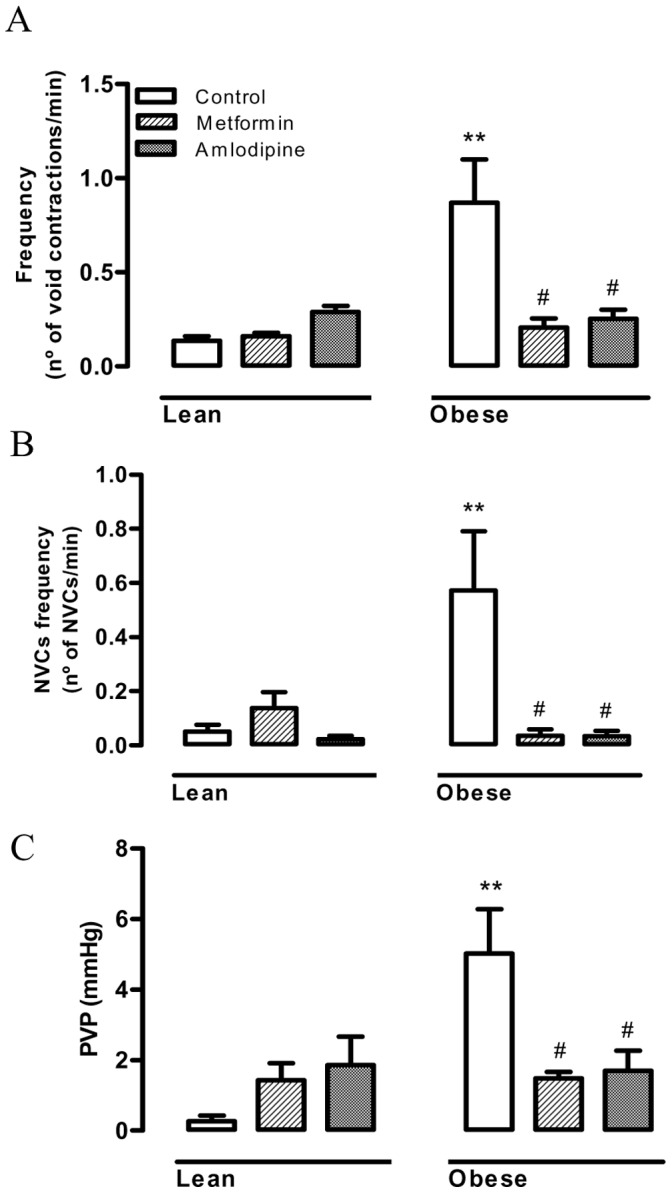
Cystometric parameters in lean and obese mice treated or not with metformin (300 mg/kg/day, 14 days) or amlodipine (25 mg/kg/day, 21 days). (A) Frequency of voiding contractions, (B) Frequency of non-voiding contractions and (C) Post-void pressure. Data represent the mean ± SEM for 5–7 mice each group. ** p<0.01 compared with control group, # p<0.01 compared with untreated obese mice.

During cystometry, lean mice showed regular micturition cycles with rare non-voiding contractions (Figure 5A). In contrast, obese mice exhibited an irregular micturition pattern (Figure 5B) and significant increases (p<0.01) in the frequency of voiding and non-voiding contractions (Figure 6 A and B; n = 5–7). The post-void pressure (PVP), which reflects the efficiency of bladder emptying, was markedly higher (p<0.01) in obese mice compared with the lean group (Figure 6C). The bladder capacity, threshold pressure, compliance and peak pressure did not significantly differ between lean and obese mice (Table 2).

**Table 2:** Values of cystometric parameters evaluated in lean and obese mice treated or not with amlodipine (25 mg/kg/day, 21 days) or metformin (300 mg/kg/day, 14 days).

	Lean mice			Obese mice		
	Untreated	Amlodipine	Metformin	Untreated	Amlodipine	Metformin
**Capacity (ml)**	0.13±0.01	0.15±0.02	0.19±0.04	0.13±0.02	0.13±0.03	0.16±0.02
**Threshold pressure (mmHg)**	3.6±0.7	5.3±0.7	4.2±0.3	4.3±0.9	4.0±0.6	5.4±0.9
**Compliance (mmHg/mL)**	0.03±0.004	0.03±0.007	0.04±0.009	0.03±0.009	0.03±0.007	0.036±0.011
**Peak pressure (mmHg)**	12.2±0.82	9.3±1.02	13.9±1.44	12.2±2.3	9.9±0.88	16.2±1.9

Results are expressed as means ± SEM from 4–7 animals in each group.

Since amlodipine normalized the overactive DSM in obese mice, we further investigated its effects on the urodynamic changes. Long-term treatment with amlodipine had no significant effects on the cystometric parameters of lean mice (n = 5–7). However, this treatment prevented the increased frequencies of voiding and non-voiding contractions, and prevented the increased PVP seen in the obese group (Figures 5C and 6). The other cystometric parameters (capacity, threshold pressure, compliance, and peak pressure) remained unchanged in amlodipine-treated mice (Table 2).

As the improvement of insulin sensitivity by chronic treatment with metformin also normalized the overactive DSM in obese mice, we next investigated the effect of this anti-hyperglycemic agent on the cystometric alterations. The increased frequency of voiding and non-voiding contractions, as well as the increased PVP in obese mice was normalized by metformin treatment (Figures 5D and 6). Metformin treatment did not significantly affect these parameters in lean mice (Figure 6). The other cystometric parameters remained unchanged in metformin-treated mice (Table 2).

### Role of Protein Kinase C (PKC) in the Enhanced Contractile-responses

**Figure 7:**
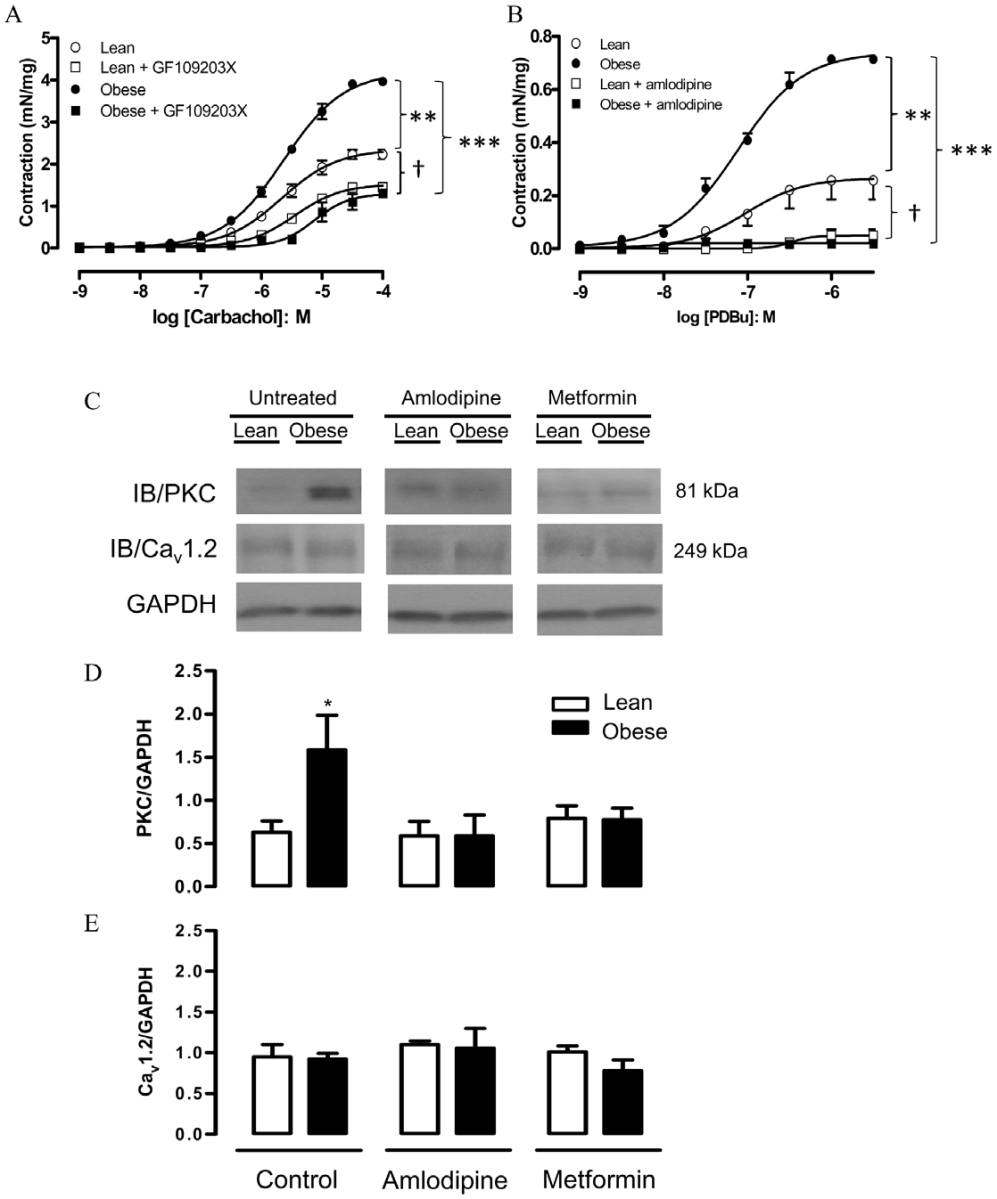
Contractile responses to carbachol (A) and phorbol-12,13-dibutyrate (PDBu; B) in detrusor smooth muscle from lean and obese mice. Responses to carbachol were performed in the presence of the PKC inhibitor GF109203X (1 µM, 30 min), whereas responses to PDBu were performed in the presence of amlodipine (3 µM). Protein expression (Western blotting) for PKC and α1 subunit of Ca_v_1.2 calcium channel in bladder tissues from lean and obese mice, treated or not with either amlodipine (3 µM, 30 min) or metformin (300 mg/kg/day, 14 days), is shown in panels C, D and E. Data represent the mean ± SEM for 4 to 7 mice each group. * p<0.05, ** p<0.01 compared with untreated lean group; *** p<0.001, † p<0.05 compared with respective amlodipine groups.

As PKC regulates Ca_v_1.2 calcium channel activity in various smooth muscle cell types, including DSM [20], we investigated the role of PKC in the enhanced DSM contractions in obese mice, by using functional and molecular approaches. The PKC inhibitor GF109203X (1 µM) normalized the enhanced carbachol-induced contractions in obese mice (Figure 7A). In addition, the PKC activator PDBu (0.001–3 µM) produced concentration-dependent contractions in strips of isolated DSM that were greater in the obese than the lean group (Figure 7B; n = 4–7; p<0.01). Prior incubation with amlodipine (3 µM) abolished the PDBu-induced DSM contractions in both obese and lean mice (Figure 7A). Similar data were obtained with chronic treatment with amlodipine (n = 4; not shown). No contractile responses to PDBu were observed in Ca^2+^-free medium (n = 3; not shown).

The PKC protein expression was significantly higher in bladder tissues from obese mice (p<0.05), and the increase in PKC expression was abrogated by amlodipine or metformin (Figure 7 C, D and E). Neither of these treatments affected the expression of PKC in the lean group. Expression of the α1 subunit of Ca_v_1.2 was equivalent in all treatment groups (Figure 7C, D and E).

## Discussion

The present study shows that high-fat fed obese mice display overactive bladder and enhanced PKC protein expression in bladder tissues that are normalized by blockade of Ca_v_1.2 and improvement of insulin sensitivity. It is likely that insulin resistance in obese mice plays a major role in the pathophysiology of this urological disorder.

The term “metabolic syndrome” describes the combination of metabolic abnormalities or risk factors for type II diabetes and cardiovascular diseases, such as central obesity, dyslipidemia, hypertension, insulin resistance and glucose intolerance [2], [28]. Among these risk factors, central obesity is regarded to be the major determinant criteria for metabolic syndrome [4]. In the model of diet-induced obesity employed here, mice exhibited increased body weight, epididymal fat mass (corresponding to central obesity in human) and insulin resistance. Previous studies have also identified dyslipidemia and impaired glucose tolerance in high-fat fed mice [22], [23]. These changes suggest that this mouse model of metabolic syndrome closely mirrors the changes in humans with metabolic syndrome / type II diabetes. Obese mice did not show increased tail-cuff pressure, excluding arterial hypertension as a cause for the present functional bladder alterations.

Epidemiological studies support a strong causal link between obesity and the development of urinary incontinence [29]. Data from our urodynamic study revealed detrusor overactivity in obese mice, as evidenced by the higher frequencies of micturition and non-voiding contractions (NVC), as well as the increased post-voiding pressure (PVP). On the other hand, the bladder capacity, threshold pressure, compliance and peak pressure did not significantly change in the obese group. This contrasts with streptozotocin-induced type I diabetes in mice, where all of these parameters are increased in comparison with control animals [21], possibly as a consequence of the enhanced urine output (diuresis) and the resulting bladder remodeling [30]. Obesity / metabolic syndrome markedly increase the risk of benign prostatic hyperplasia (BPH) that is in turn a risk factor for detrusor overactivity resultant from bladder outlet obstruction [31]. High fat-fed insulin-resistant Sprague Dawley rats exhibit prostate enlargement that is prevented by treatment with the anti-hyperglycemic agent pioglitazone [32]. Therefore, in our study, whether the increased PVP and poor bladder emptying efficiency seen in obese mice reflect a failure to compensate for enhanced urethral resistance due to prostate enlargement requires further studies [32].

Urinary bladder function is regulated by a complex interaction of efferent and afferent fibers from the autonomic nervous system and somatic innervations [33]. The neurogenic contractions of the bladder mainly reflect the release of the excitatory transmitter acetylcholine (ACh) from parasympathetic fibers. The bladder contains all mAChR subtypes, but the M_3_ receptor is responsible for the urinary bladder contractions [34]. The excitatory transmitter ATP, through ionotropic P2X1 receptors, also mediates part of the atropine-resistant neurogenic bladder contractions under normal and pathophysiological conditions, although to varying extents across species from rodent to man [35]. There is also a muscarinic- and purinergic-resistant neurogenic component that has not yet been fully characterized [36], [37]. In our study, neurogenic- and carbachol-induced DSM contractions were greater in obese mice compared with lean mice, whereas no significant differences for the purinergic P2X1 agonist α,β-methylene ATP were found.

Muscarinic M_3_ receptors interact with Gq to elicit phosphoinositide hydrolysis and generation of the second messenger inositol-1,4,5-trisphosphate (IP_3_), which activates the IP_3_ receptor to release Ca^2+^ from internal stores [34]. Muscarinic agonist-induced contractions have also been shown to partly depend on Ca^2+^ entry through Ca_v_1.2 channels, as evidenced in animal and human bladders under physiological conditions [38]–[42]. High levels of extracellular K^+^ depolarize the cell membrane and activate Ca_v_1.2 channels, resulting in increased inward movement of Ca^2+^, which in turn activates contractile proteins [43]. In smooth muscle tissues, dihydropyridine Ca^2+^ channel blockers inhibit the increase in [Ca^2+^]_i_ induced by high K^+^ or extracellular CaCl_2_. Our findings that DSM strips obtained from obese mice show greater contractile responses to both receptor–dependent (carbachol) and –independent agents (KCl and extracellular CaCl_2_) strongly suggest that enhanced extracellular Ca^2+^ entry via Ca_v_1.2 channels plays a critical role in the overactive DSM in obese mice. This is reinforced by our data showing that pretreatment with the dihydropyridine Ca^2+^ channel blocker amlodipine fully reversed the enhanced contractile responses to carbachol, KCl and CaCl_2_ in obese mice. The urodynamic alterations in the obese group were also reversed by treatment of mice with amlodipine, which is consistent with the reversal of the enhanced DSM contractions in vitro to carbachol, KCl and extracellular CaCl_2_ by amlodipine.

The signaling cascade for M_3_ receptors in smooth muscle also involves generation of DAG leading to PKC activation and inhibition of myosin light-chain (MLC) phosphatase, thus enhancing the contractile response [43]. Dysfunctions of the DAG–PKC pathway have been associated with vascular abnormalities in diabetic and insulin resistant states [44]. In the present study, we have performed concentration-response curves to carbachol in the presence of the PKC inhibitor GF109203X, and found that enhanced DSM contractions in the strips taken from obese mice were nearly normalized by this inhibitor. Additionally, the PKC activator PDBu produced concentration-dependent DSM contractions that were markedly greater in the strips taken from obese mice, an effect nearly abolished by amlodipine. This is consistent with the higher PKC protein expression in the bladder tissues of obese mice. Our data that the α1 subunit of Ca_v_1.2 channel expression remained unchanged between groups together with functional data suggest that overactive DSM in obese mice takes place through elevated Ca_v_1.2 channel activity, increasing the extracellular Ca^2+^ influx. However, the lack of a direct measurement of Ca_v_1.2 activity or extracellular Ca^2+^ influx by electrophysiological studies in the bladder limits our comprehension about the observed phenomenon. We cannot ascertain, for instance, if changes in each of the component of the M3/PKC/Cav1.2 axis act independently or as a signaling pathway to determine the overactive DSM. Ca_v_1.2 channels have been suggested to serve as an anchoring target for PKC during translocation of this protein to the cell membrane after agonist stimulation [20]. The increased carbachol-induced bladder contractions observed under *in vitro* hyperglycemic conditions has also been associated to rho-kinase-mediated increased PKC activity [45]. Interestingly, in our study, amlodipine normalized the PKC protein levels in bladder tissue of obese mice. One may speculate that inactivation of Ca_v_1.2 channels results in downregulation of PKC expression, which is consistent with a previous study showing that amlodipine decreases the phosphorylation and activity of PKC in cultured human endothelial cells stimulated with the PKC activator PMA [46].

Metformin is a first-line pharmacological treatment for patients with type II diabetes mellitus because of its favorable overall profile, including its glucose-lowering ability, weight-neutral effects, and low risk of hypoglycemia. In our study, oral treatment of obese mice with metformin normalized the insulin resistance, as well as the *in vitro* and the *in vivo* (cystometry) bladder dysfunction. Moreover, metformin reversed the enhanced PKC levels in the bladder tissues from obese mice. Accordingly, a previous study has shown that PKC-θ knockout mice are protected from fat-induced insulin resistance in skeletal muscle [47]. Our findings that *in vitro* incubation of DSM strips with metformin does not alter the overactive DSM indicate that normalization of bladder function resultant from the long-term treatment with this anti-hyperglycemic agent is secondary to the improvement of insulin sensitivity, rather than through a direct effect on the contractile machinery. Atypical and conventional PKC isoforms are classically related to mechanisms that contribute to insulin resistance in peripheral tissues responsible for glucose disposal and energy homeostasis [48]. Our present results evidence that PKC activation induced by high fat feeding plays a role in a very specific complication associated with insulin resistance. Thus, our data suggest that PKC activity plays an important pathological role that is beyond the impairment glucose homeostasis control. In agreement with our findings, endothelial insulin resistance and vascular hyperactivity in high fat diet fed mice was recently described to be ameliorated by PKC inhibition [44], [49], suggesting that the mechanism presently described is not specific for the bladder.

Obese mice treated with metformin remained overweight, suggesting that insulin resistance and subsequent hyperglycemia, rather than obesity, is the main cause of bladder overactivity. Although metformin reduces body weight and waist circumference in humans [50], no such effect has been reported in animals treated chronically with this agent [51]. Interestingly, in bladder dysfunction in female obese Zucker rats, it was the presence of chronic obesity rather than the onset of diabetes that led to impaired bladder function [13].

In summary, the present study shows that the contractile and urodynamic alterations as well as the enhanced PKC expression in bladder tissues seen in high-fat fed obese mice are normalized by blockade of Ca_v_1.2 and improvement of insulin sensitivity. Up-regulation of PKC in obese mice is likely to simultaneously mediate the insulin resistance and hence overactive bladder.
